# Grappling the High Altitude for Safe Edible Bamboo Shoots with Rich Nutritional Attributes and Escaping Cyanogenic Toxicity

**DOI:** 10.1155/2013/289285

**Published:** 2013-11-19

**Authors:** Sayanika Devi Waikhom, Bengyella Louis, Chandradev K. Sharma, Pushpa Kumari, Bharat G. Somkuwar, Mohendro W. Singh, Narayan C. Talukdar

**Affiliations:** ^1^Institute of Bioresources and Sustainable Development (IBSD), Takyelpat, Imphal, Manipur 795001, India; ^2^Department of Biochemistry, University of Yaoundé I, BP 812, Yaoundé, Cameroon; ^3^Department of Biotechnology, Burdwan University, Golapbag More, West Bengal 713104, India; ^4^AJC Bose Indian Botanic Garden, Botanical Survey of India, Botanic Garden, Howrah 711103, India

## Abstract

Consumption of bamboo species with high level of total cyanogenic content (TCC) in Asia by many ethnic groups is significantly associated with food poisoning and occasionally Konzo (a neurological disorder). Adequate characterization of edible bamboo species with low level of TCC and high nutritious attributes is required for consumer's safety as well as for the conservation of the gene pool. Here, we employed morphological descriptors, atomic absorption spectrophotometer, RAPD, and trnL-F intergenic spacer to characterize 15 indigenous edible bamboo species of north-east India. The study indicates that morphologically and genetically evolved edible bamboo species having large and robust bamboo-shoot texture and growing at low altitude contain high level of TCC, low antioxidant properties, and low levels of beneficial macronutrients and micronutrients. Importantly, *Dendrocalamus* species are shown to be rich in TCC irrespective of the growing altitude while *Bambusa* species are found to have moderate level of TCC. The findings clearly demonstrated that *Chimonobambusa callosa* growing at high altitude represents safe edible bamboo species with nutritious attributes.

## 1. Introduction

Bamboo shoots are popular traditional food delicacies which are consumed as fresh, fermented, or canned in many South-East Asian countries. Cyanogenic glycosides are inherently produced in cyanogenic plants as defence arsenals and abundantly produced in bamboo shoots in the form of taxiphyllin [1, 2]. High intake of cyanogenic glycosides is life threatening and significantly associated with neurological disorder called Konzo [3]. Under optimal conditions, lactic acid from fermentation of bamboo shoot reduces total cyanide content (TCC) [4]. Nonetheless, better results can be achieved if the initial starting material is poor in TCC. Therefore, ingestion of fresh or inappropriately fermented bamboo shoots can lead to cyanide poisoning.

Irrespective of toxicity, some young edible bamboo shoots (≤30 days) possess enormous nutritious potentials such as high fibre content with antioxidant and antitumor properties [5, 6]. Consequently, the demand for bamboo shoots is high and farmers are often challenged to match supply in both quality and quantity. The magnitude of this increasing demand is exacerbated by nondomestication of edible bamboo species. As a result, nonedible bamboo (or poisonous) shoots are made available in the market which are harmful to consumers. In order to effectively outwit this anthropogenic pressure, a holistic approach is required to identify the species with rich nutritional attributes for safe human consumption and domestication.

Biochemical studies on antioxidant activity, cyanogenic glycoside content, and nutrient content have been reported for a few species [5, 7–9], without correlation to the altitudes of sample collection, equally lacking morphological and molecular characterization. Based on previous work, it is difficult to sort out edible bamboo species, requiring low-processing capital inputs to eliminate the poisonous components. Here, we exploit the geographic positioning of edible bamboo species in a dynamic biota and their morphological descriptors, RAPD and trnL-F intergenic spacer, to study the interrelatedness of coevolved species of genera *Bambusa*, *Dendrocalamus*, *Chimonobambusa*, *Schizostachyum*, and *Melocanna*. We also exploit the geographic positioning of same bamboo species to shed light on how altitude influences the valuable nutritional attributes of bamboo shoots.

## 2. Material and Method

### 2.1. Plant Material

Fifteen species of edible bamboo-shoots belonging to the genera *Bambusa*, *Dendrocalamus*, *Chimonobambusa*, *Schizostachyum*, and *Melocanna* were collected from different altitudes of Manipur, India (23°47′–25°41′ NL; 92°58′–94°47′ EL), during July-August of 2009-2010. This region often receives an average rainfall of 1320 ± 3 mm and temperature of 23 ± 3°C during the months of July-August. This group of samples was used for morphological characterization and authenticated by the Botanical Survey of India (BSI), Kolkata. The voucher specimens were deposited at the Central National Herbarium in BSI for future references (Table 1). For biochemical analysis, 30-day-old bamboo-shoots (from the day of emergence) from the field were collected in 2009, 2010, and 2011 following routine sampling. Subsequent to harvest, the bamboo-shoots were directly frozen in liquid nitrogen in a thermocol box and transported to the laboratory where they were stored at −80°C for downstream analysis. This group of samples was collected at sunset and the soil pH for the sites of collection was determined as previously described [10].

### 2.2. Morphological Analysis

To evaluate the morphological interrelatedness among the edible bamboo species, we used 35 morphological descriptors based on 11 culm types, 13 culm-sheath types, and 11 leaf types (Table S1 available online at http://dx.doi.org/10.1155/2013/289285). The morphological data were analysed using NTSYS-PC version 2.2 [11]. Simple coefficient matching was performed using SIMQUAL option for generating dataset similarity matrix [12]. The best dendrogram was computed with Unweighted Pair-Grouped Method Arithmetic Averages (UPGMA) [13]. All the samples used in this study were scored in triplicate.

### 2.3. DNA Extraction

To characterize at molecular level, genomic DNA was isolated as described by Aras et al. [14]. The quantity and quality of DNA were checked on BioSpec NanoDrop spectrophotometer (Thermo Scientific, USA) and on 0.8% w/v agarose gel electrophoresis, respectively.

### 2.4. Random Amplified Polymorphic DNA (RAPD) Analysis

PCR was performed using standard RAPD PCR kit (Invitrogen, USA) in a volume of 50 *μ*L in C1000 Touch Thermal Cycler (BIO-RAD, USA). The run was programmed as follows: initial denaturation at 95°C for 5 min, followed by 35 cycles of amplification (95°C for 1 min, 37°C for 1 min, and 72°C for 2 min) and a final extension at 72°C for 7 min. Amplicons were profiled on a 1.8% agarose gel electrophoresis and revealed with ethidium bromide in Gel Doc-it^2^ Imager (UVP Co., Ltd.). The primers used for this analysis are represented (Table S2). Polymorphism was scored as 1/0 (presence or absence) to generate a primary binary matrix. The primary binary matrix was used for producing similarity data using Jaccard's similarity coefficient [15] and computed using UPGMA. This analysis was performed in NTSYS-PC software [11].

### 2.5. Analysis of trnL-F Intergenic Spacer

In order to authenticate the edible bamboo species, the trnL-F region was amplified using the primers set (forward: 5′-ggttcaagtccctctatccc-3′; reverse: 5′-atttgaactggtgacacgag-3′) as described in Taberlet et al. [16]. PCR products of about 350–400 bp were purified and sequenced in ABI370X1 Cycler Sequencer (ABI, USA) using the same set of primers. Sequences were automatically trimmed and assembled in DNAbaser 3.5.3 software (http://www.dnabaser.com/). Following annotation, sequences were assigned to molecular species based on 98–100% sequence similarity threshold in the GenBank (http://www.ncbi.nlm.nih.gov/) and in accordance with morphological descriptors. The sequences are available in GenBank as accessions JX564900 (*Bambusa manipureana*), JX564901 (*Bambusa nutans*), JX564902 (*Dendrocalamus giganteus*), JX564903 (*Dendrocalamus hamiltonii*), JX564904 (*Dendrocalamus hookeri*), JX564905 (*Dendrocalamus manipureanus*), JX507131 (*Bambusa oliveriana*), JX507132 (*Bambusa tulda*), JX507133 (*Melocanna baccifera*), JX507134 (*Schizostachyum dullooa*), KC013282 (*Chimonobambusa callosa*), KC013285 (*Bambusa cacharensis*), JX564906 (*Bambusa *sp.), JX564907 (*Bambusa *sp.), and KC013288 (*Bambusa tuldoides*), respectively.

Sequences were aligned using Clustal omega program [17]. The program BioEdit [18] was used to assess the nature of variability and the entropy of the alignment among the species. The program TOPALi v.2.5 [19] was used to select the best substitution model and the best method for phylogenetic tree reconstruction based on Akaike Information Criterion, corrected AICc_1_ and AICc_2_, and Bayesian Information Criterion (BIC). Phylogenetic analysis was performed using the maximum likelihood (ML) method. Hasegawa-Kishino-Yano nucleotide substitution model was used [20]. The initial trees for the heuristic search were obtained automatically as follows. When the number of common sites was <100 or less than one-fourth of the total number of sites, the maximum parsimony method was used; otherwise BIONJ method with Monte Carlo localization (MCL) distance matrix was used. A discrete Gamma (+G) distribution was used to model evolutionary rate differences among sites (5 categories (+G, parameter = 200.00)). The analysis was performed in MEGA5 [21].

### 2.6. Determination of Total Cyanide Content

To determine the poisonous potential of each species, the total cyanide content (TCC) was evaluated using the picrate method as described in Bradbury et al. [22] with some modifications. Firstly, bamboo shoot sheaths were removed and the innermost edible portion was measured using a ruler and a slide calliper. The full length was divided into three equal parts, that is, the tip, the middle, and the base. Woody bamboos generally grow rapidly and their shoots are often eaten young [4–6]. Moreover, based on preliminary findings using 10-, 20-, and 30-day-old bamboo shoots (data not shown), revealed that TCC and other nutritional parameters studied insignificantly varied with only 30-day-old sample. Cogently, only 30-day-old bamboo shoots were used for the biochemical analysis. The standard curve for determination of HCN was established using NaCN solution as follows: 5 mL of alkaline picrate solution (1.4 g of picric acid in 2.5% Na_2_CO_3_) and 5 mL of NaCN solution (181 mg of NaCN in 1 L sterile milli-Q water) were pooled together to obtain 100 *μ*g HCN/mL and heated for 5 min in boiling water. Volumes of 0.1, 0.2, 0.4, 0.6, and 0.8 and 1 mL of the resultant NaCN alkaline picrate solution were adjusted to 5 mL with sterile milli-Q water to obtain 5, 10, 20, 30, 40, and 50 *μ*g HCN, respectively.

### 2.7. Antioxidant Activity Estimation

100 g of sliced bamboo shoot was boiled in 300 mL of double distilled water for 2 h at 100°C. The crude extract was filtered through Whatman no. 42 filter paper and concentrated in a rotary evaporator at 100°C. The solid residues were stored at 4°C till used. The scavenging effect of 2,2-diphenyl-1-picrylhydrazyl (DPPH) free radical was assayed as previously described in Mensor et al. [23]. L-Ascorbic acid (Sigma, USA) was used as reference antioxidant control. One mL of 0.3 mM DPPH ethanolic solution was added to each sample at different concentrations of 20 *μ*g, 50 *μ*g, 100 *μ*g, 200 *μ*g, and 400 *μ*g. The mixture was vortexed for 1 min and then left to stand at room temperature in the dark. After 30 min, absorbance was read at 517 nm in UV1700 spectrophotometer (Shimadzu, USA). The scavenging activity of DPPH free radical was calculated using the following equation:

(1) $$\begin{array}{c} \text{scavenging}\;\text{activity}\;\;\left(\%\right)=100\times \frac{\left[A_{C}-A_{S}\right]}{A_{C}}. \end{array}$$

*A*_*C*_ is the absorbance of the control reaction (containing all reagents except for the test compound) and *A*_*S*_ is the absorbance of the test compound. The inhibition concentration (IC_50_) is defined as the amount of extract required to reduce free scavenging activity by 50%. The IC_50_ values were obtained from the inhibition curve by extrapolation. 

### 2.8. Estimation of Macro- and Micronutrients

Total nitrogen contents were determined through digestion and distillation of dry bamboo shoots in Kel-Plus digestion system (Pelican, India) according to AOAC [24] protocol. Crude protein was calculated as Kjeldahl N × 6.25 based on the assumption that nitrogen (N) constitutes 16.00% of a protein. The element contents such as potassium (K), sodium (Na), calcium (Ca), magnesium (Mg), copper (Cu), iron (Fe), and zinc (Zn) were estimated using atomic absorption spectrophotometer (AAS) (Perkin Elmer, USA). 500 mg of dried weight (d.w) bamboo shoot powder was digested in 10 : 4 : 1 (HNO_3_ : HClO_4_ : H_2_SO_4_). The digested sample was appropriately diluted with sterile deionized water and filtered with Whatman no. 42 filter paper. All the elements were analyzed with appropriate multielement hollow cathode lamps (Lumina Lamp, Perkin Elmer) against a standard reference solution for AAS (Accutrace Reference Standard, USA). Phosphorus was estimated by absorbance measurement at 420 nm of the vanadomolybdophosphoric heteropoly complex formed in the digestate [25]. Cellulose (Ce) content was estimated at 620 nm using cold anthrone reagent method [26].

### 2.9. Statistical Analysis

One-way analysis of variance (ANOVA) was implemented to compare the means of different treatments. The differences between individual means were tested using the least significant difference (LSD). Computation was performed in SPSS software (version 22.0, SPSS Inc., Chicago, USA). The relationship between the different biochemical attributes and 13 edible bamboo species was analysed using principal component analysis (PCA). PCA on standardized data was performed in NTSYS-PC version 2.2 [11]. Principal components with eigenvalues (*ε* > 1.00) were selected and correlation values (*r* > 0.30) were considered as relevant for the PCA. Three genera underrepresented in the study set of 15 were excluded from principal component analysis.

## 3. Results

### 3.1. Morphological Analysis

**Figure 1:**
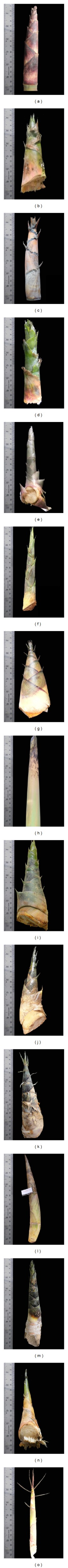
Morphological features of 30-day-old bamboo shoots imaged with Nikon Coolpix S6200 and the nomenclature as submitted in the Genbank NCBI nucleotide database: (a) KC013282/*C. callosa*, (b) KC013285/*B. cacharensis*, (c) JX564900/*B. manipureana*, (d) JX564901/*B. nutans*, (e) JX507132/*B. tulda,* (f) JX507131/*B. oliveriana*, (g) JX564902/*D. giganteus*, (h) JX564903/*D. hamiltonii*, (i) JX564906/*Bambusa* sp., (j) JX564904/*D. hookeri*, (k) JX564905/*D. manipureanus*, (l) JX564907/*Bambusa* sp., (m) KC013288/*B. tuldoides*, (n) JX507133/*M. baccifera*, and (o) JX507134/*S. dullooa*.

**Figure 2:**
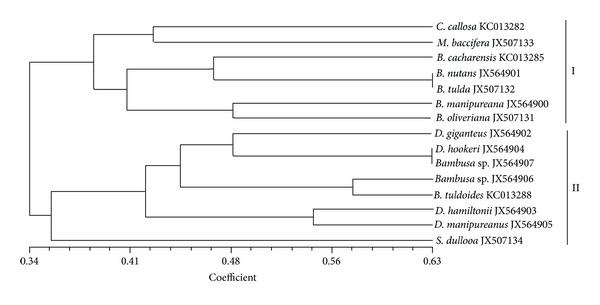
A dendrogram based on morphological descriptors showing the relationship between 15 edible bamboo species generated in NTSYS-PC software computed based on simple matching coefficient [11].

Of the 15 identified edible bamboo species studied, only 13 were morphologically identified at the species level and deposited in BSI, Kolkata (Table 1). The morphological characteristics of the young (<30 days) bamboo shoots are depicted in Figure 1. In the study set, bamboo shoots of JX564902/*D. giganteus*, JX564903/*D. hamiltonii*, JX564904/*D. hookeri*, and JX564905/*D. manipureanus* were generally deep green, broad based with robust texture (Figures 1(g), 1(h), 1(j), and 1(k)). An overall strong cophenetic correlation coefficient of 0.70 based on morphological characteristics (Table S1) was obtained, indicating a faithfully constructed dendrogram (Figure 2). The bamboo species clustered into two main clades (I and II) with JX507134/*S. dullooa* evolving in a polyphyletic pattern. The clustering pattern was significantly affected by the morphological characteristics such as colour, shape, and presence of hairs in culm sheaths covering the shoots.

### 3.2. RAPD Analysis

**Figure 3:**
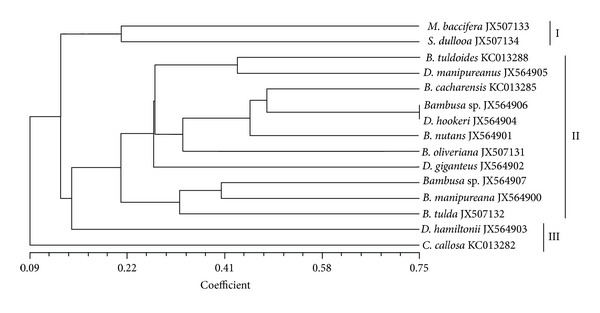
A dendrogram based on Jaccard's similarity coefficient obtained from RAPD data showing the relationship between 15 edible bamboo species.

High level of polymorphism was observed among the 15 species based on the 9 primer sets (Table S2). A strong co-phenetic coefficient of 0.77 based on UPGMA analysis was obtained. A dendrogram (Figure 3) based on band differences revealed three clades (I, II, and III) with *C. callosa* forming an out group. In this analysis, RAPD evidence of JX564903/*D. hamiltonii* followed a polyphyletic evolutionary pattern (clade III). The gel profile (Figure S1) provides evidence of JX507131/*B*.* oliveriana* (lane 7) *and* JX564907/*Bambusa* sp. (lane 15) with higher numbers of dominant characters than those of other species as reflected in the banding pattern. 

### 3.3. DNA Sequence Analysis

**Figure 4:**
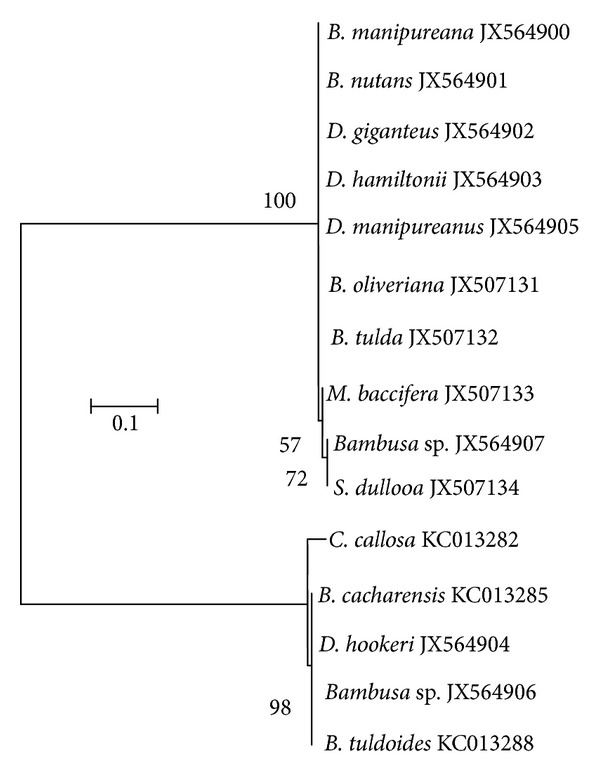
A maximum likelihood tree based on Hasegawa et al. [20] nucleotide substitution model. The tree is drawn to scale, with branch lengths measured in the number of nucleotide substitutions per site. Evolutionary analyses were conducted in MEGA5 [21].

Based on DNA sequences, the estimated model parameters were base frequencies (A = 25%, T/U = 25%, C = 25%, and G = 25%) and substitution model [T/U↔A] = 7.10, [C↔A] = 7.10, [G↔A] = 10.80, [C↔T/U] = 10.80, [G↔T/U] = 7.10, and [G↔C] = 7.10. The estimated transition-transversion bias (*R*) ratio was at 0.76. The overall mean Tajima-Nei [27] evolutionary distance among the species was 0.51. In the sequence set, the entropy of the alignment (Figure S2) showed 164 patterns (out of a total of 491 sites) and 276 sites were without polymorphism (56.21%). A maximum likelihood tree with the highest log likelihood (−445.63) supported by 1000 bootstrap test of replicates showing two main clades (I and II) was generated (Figure 4). It was observed that *Dendrocalamus* spp. formed a close complex relationship with *Bambusa *spp. The tree without branch swapping evidence *C. callosa* has evolved differently from the rest of the edible bamboo species, thus, forming an out group.

### 3.4. Total Cyanide Content

The level of TCC in all the species varied from 300 to 2604 ppm (for the tip portion), 210 to 2243 ppm (for the middle portion), and 199 to 920 ppm (for the basal portion). These significant differences (at *P* < 0.05) in toxicity level suggest that bamboo shoot tips are generally toxic. On the contrary, a low level TCC was observed in *C. callosa* collected from high altitude in all the studied segments, that is, the tip, the middle, and the base portions of the bamboo shoots (Table 2). Often, all bamboo shoots collected from low altitude were rich in TCC, except for *M. baccifera *and *B. manipureana*, with respect to *C. callosa* collected from high altitude. Overall, all *Dendrocalamus* species, growing at either high altitude (>700 m) or low altitude (<400 m), were rich in TCC in comparison with other genera (Tables 1 and 2).

### 3.5. Antioxidant Activity

**Figure 5:**
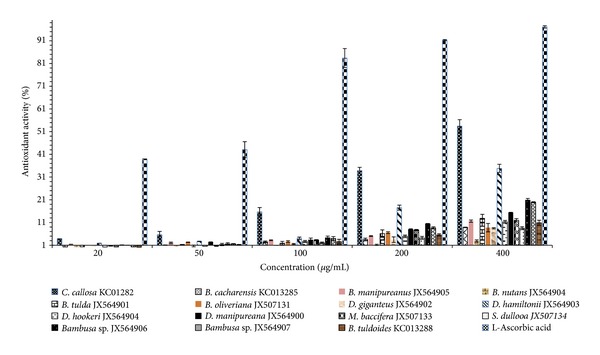
Antioxidant activity of 15 bamboo shoot extracts against DPPH free radical with respect to L-ascorbic acid as reference antioxidant agent.

Bamboo shoot extract of *C. callosa* showed the highest significant antioxidant activity (of 53.46%, *P* < 0.05) at 400 *μ*g/mL of DPPH (Figure 5), whereas the lowest antioxidant activity (of 2.90%, *P* < 0.05) was obtained with extract of *B. nutans*. Akin to this pattern, the half-inhibition concentration (IC_50_) obtained by linear regression analysis showed a significant variation from 0.09 mg/L for *C. callosa* to 1.83 mg/mL for* B. nutans *(Table 2). Among the three *Dendrocalamus* species obtained at altitude (>700 m), *D. giganteus *possessed the least scavenging activity with an IC_50_ value of 0.60. L-Ascorbic acid investigated under the same conditions had an IC_50_ value of 0.003 mg/mL; implying that bamboo shoots have a moderate antioxidant activity (Table 2). Based on IC_50_, *D. giganteus* is 30-fold poorer in antioxidant activity than an equivalent weight of L-ascorbic acid. When put together, our results suggest that *Dendrocalamus * species collected at high or low altitude possess low antioxidant attributes (Tables 1 and 2).

### 3.6. Estimation of Macro- and Micronutrients

Atomic absorption spectrometry (AAS) data revealed that bamboo shoots are generally rich in nitrogen, phosphorous and contain moderate amount of calcium. Remarkably, the highest nitrogen (1153 mg/100 g d.w) and phosphorous (1154 mg/100 g d.w) content was found in *C. callosa*. By contrast, the lowest nitrogen (673 mg/100 g d.w) and phosphorous content (70 mg/100 g d.w) was observed in *D. giganteus*. Among the important macronutrients studied (N, P, and K), all the bamboo shoots were rich in potassium and poor in sodium (Table 3). Of all the microelements assessed, iron was significantly present in *B. tulda* at the rate of 25.80 mg/100 g dry weight (d.w) and was lowest in *D. hookeri* at the rate of 6.08 mg/100 g d.w. We observed a high level of zinc in *B. tulda* at the rate of 15 mg/100 g d.w and its lowest level in *D. hookeri* at the rate of 3.03 mg/100 g d.w. We found a significant amount of copper only in *C. callosa* at the rate of 6.12 mg/100 g d.w (Table 3).

### 3.7. Principal Component Analysis (PCA)

**Figure 6:**
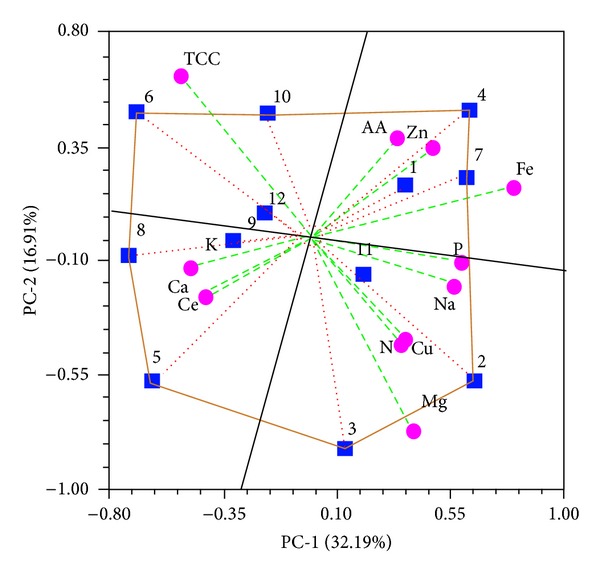
PCA polygonal biplot analysis showing the interrelatedness of nutritional attributes of 12 edible bamboos species. The numbers represent bamboo species and the vectors are biochemical traits: KC013285/*B. cacharensis* (1), JX564900/*B. manipureana* (2), JX564901/*B. nutans* (3), JX507132/*B. tulda* (4), JX507131/*B. oliveriana* (5), JX564902/*D. giganteus* (6), JX564903/*D. hamiltonii* (7), JX564906/*Bambusa* sp. (8), JX564904/*D. hookeri* (9), JX564905/*D. manipureanus* (10), JX564907/*Bambusa* sp. (11), and KC013288/*B. tuldoides* (12). AA: antioxidant activity, Ca: calcium, Ce: cellulose, Cu: copper, Fe: iron, K: potassium, Mg: magnesium, N: nitrogen, Na: sodium, P: phosphorous, TCC: total cyanide content, and Zn: zinc.

Using graphical approaches to study biological problems can provide an intuitive picture or useful insights for analysing complicated relationship in large data set [28], as demonstrated by many previous studies on a series of important biological topics, such as enzyme-catalysed reactions [29–31], inhibition of HIV-1 reverse transcriptase [32], drug metabolism systems [33], and using Wenxiang diagram [34] to study protein-protein interactions [35, 36]. The interrelatedness of all the biochemical traits studied and their relationship with the bamboo species based on PCA generated four principal components: PC-1, PC-2, PC-3, and PC-4. The four principal components with eigenvalues (*ε* > 1.00) accounted for 72.25% of the total variation in nutritional attributes (Table S3). A positive correlation coefficient (*r* > 0.30) was observed in PC-1 among phosphorous, iron, sodium, copper, magnesium, zinc, nitrogen, and antioxidant activity, accountable for 32.19% (*P* < 0.05) variation in nutritional content. Interestingly, we observed that PC-2 and PC-3 were highly associated with nitrogen, cellulose, magnesium, copper, and potassium (16.91% variation, *P* < 0.05) and total cyanide content, calcium, cellulose, zinc, and copper (14.50% variation, *P* < 0.05), respectively. In PC-2 and PC-3, no significant correlation was observed among nutritional attributes. PC-4 was associated with cellulose, antioxidant activity and accounted for 11.65% (*P* < 0.05) net variation in nutritional attributes. The interrelatedness between the nutritional attributes is represented in a polygonal biplot (Figure 6) as previously described [37].

The polygonal biplot (Figure 6) is divided into four sectors by values 2, 3, 4, 5, 6, 7, 8, and 10 with the vertex representing the species. In this representation, the species are the best or the poorest contributing for some or all of the biochemical traits [38], depending on the length of the vector lines from the origin. The biplot indicates that *B. manipureana* (from 240 m altitude) and *B. nutans* (from 226 m altitude) had the highest magnesium (Mg), copper (Cu), and nitrogen (N) denoted by 2 and 3, respectively. Similarly, *B. tulda* (from 226 m altitude) *and D. hamiltonii *(from 358 m altitude) had the highest value for antioxidant activity (AA), iron (Fe), magnesium (Mg), and zinc (Zn) denoted by 7 and 4, respectively. On the other hand, JX564906/*Bambusa* sp. (from 770 m altitude) and *D. giganteus* (from 803 m altitude) had the highest total cyanide content (TCC) denoted by 6 and 10, respectively. The numbers 5 and 8 representing *B. oliveriana* (from 728 m altitude) and *D. hookeri* (from 770 m altitude) were found to be tightly linked with high level of calcium (Ca), cellulose (Ce), and potassium (K), respectively. In relative terms, PCA polygonal biplot intuitively evidences that *D. giganteus* (from 803 m altitude) is rich in TCC. *C. callosa, M. baccifera, and S. dullooa* were excluded from PCA biplot analysis for reason of underrepresentation of the genera. 

## 4. Discussion

For any meaningful differentiation of edible bamboo species from poisonous ones, the delineating parameters must show a low level of conflicting signals. Using morphological descriptors as discerning tools to categorize the edible bamboo species, the clustering pattern was often significantly affected by dominant morphological characteristics such as colour, shape, and presence of hairs in the culm sheath covering shoots (Figure 1). Considering that most bamboo species and their shoots are green in colour, this produces conflicting signal and renders thought-provoking judgements. This is challenging because the homogeneity and conflicting heterogeneity among species are time consuming and require a high level of expertise. However, the benefit of morphological descriptors is that it permits the initial discernment of poisonous bamboo shoot (which is usually large and has robust-deep green colour) from less toxic bamboo shoots. 

Although a less efficient technique, the benefit of RAPD analysis to delineate bamboo species is its rapidity and cost effectiveness. Nevertheless, without any public database for RAPD data, the technique was not effective to barcode our edible bamboo species. The dendrogram generated by RAPD and morphological descriptors had similarity: a cophenetic coefficient is greater than 0.5 but differs by the interspecies clustering pattern. Nonetheless, RAPD discriminated JX564903/*D. hamiltonii* as a polyphyletic evolving species in the data set. This might be a false positive grouping since RAPD markers are dominant and cannot discriminate if a DNA fragment is amplified from a homozygous locus or a heterozygous locus. By contrast, the species were discriminated by point mutations observed at the trnL-F intergenic spacer region (Figure S3). This phenomenon of low variation can possibly be explained by the unique asexual reproductive life cycle and also the high rate of transversion transition at the trnL-F locus. Thus, using Kumar and Gadagkar [39] disparity test of substitution pattern homogeneity rate, we showed that variations are generally low at the trnL-F intergenic spacer region (Figure S4). Nonetheless, based on two molecular tools used in this study, we found that *C. callosa* collected at high altitude (1843 m) had undergone a unique evolving pattern (Figures 3 and 4). Evidently, DNA sequence (Figure S3) shows that *C. callosa* has suffered less from point mutation in comparison with *Bambusa* species, *Schizostachyum* species, *Dendrocalamus* species, and *Melocanna* species.

Undoubtedly, since few bamboo species have been adapted for consumption through ancestral customs and not domesticated for extensive farming, no proper study has been conducted to identify low level TCC species vis-à-vis their geographical positions in a dynamic biota. In this era of high demand for bamboo shoots, irrespective of the health risk (such as Konzo), identifying species with nutritious attributes, nonetheless, with low level TCC is indispensable. A holistic approach encompassing morphological and molecular tools which are required, considering the three barcoding tools used in this study, showed different interspecies clustering pattern. From the present data, grappling the high altitude for safe edible bamboo species allowed for low input capital for eliminating TCC during bamboo shoot processing. Nonetheless, prolonged and acute intake of diet containing cyanogen derivatives could lead to death; exacerbate goitre, cretinism in iodine deficient regions, mental confusion, irreversible paralysis of legs, and ultimately the neurological disorder of Konzo [3, 39, 40]. Intriguingly, an optimal cooking procedure (at 98–102°C for 148–180 min) has shown to reduce cyanogens by 97% in *D. giganteus* [41, 42]. Since, only *D. giganteus* was studied [42], this cooking protocol might not be applicable for other bamboo species because the binding of taxiphyllin may vary from species to species and even at the genus level.

Accordingly, the safest procedure for consuming bamboo shoots might only be by using species with low TCC as starting material. Previously, Haque and Bradbury [9] showed that the total cyanide content decreased from the tip to the base of bamboo shoots which agreed with the present study, except for the *Dendrocalamus* species which further showed significant variability, irrespective of the altitude of collection. This suggests that the *Dendrocalamus* species should most probably not be consumed or considered at all as edible bamboo. Although cyanogenic, some succulent bamboo species are shown to possess a free radical scavenging activity which is considered as good food. Explicitly, some species showed appreciable amount of antioxidant activity except for the *Dendrocalamus* species, further confirming their low nutritious value as a whole. Recently, Park and Jhon [7] evaluated the antioxidant activities of *Phyllostachys pubescens* and *Phyllostachys nigra* only and concluded that the parameter varies between the species. Here, using a larger data set we showed that there is an interspecific variation in antioxidant properties of bamboo shoots collected at different altitudes. 

The recommended dietary allowance (RDA) intake for magnesium (Mg) is 320 mg for female adults and 400–420 mg for male adults [43]. Consequently, 100 g of the bamboo shoots (except the *Dendrocalamus* species) can supply 28–75% of potassium, 1–4% of sodium, 10–22% of phosphorous and 22–56% of magnesium for females and 16–43% for adults, respectively. The RDA of iron for pregnant women is 27 mg, and thus 100 g of the bamboo shoots can provide 26–96% RDA for pregnant women. Equally, 100 g of the bamboo shoots also contained 28–136% of zinc and 2–7% of copper required as per RDA [43]. Based on our studies, *C. callosa* provides more of these beneficial RDA intakes of micro- and macronutrients as well as substantial antioxidant activity. Plants with antioxidant properties have been shown to be important natural antitumor and antimutagenic reserves [6, 44]. When put together, results of mineral content obtained in this study agreed with the previous quantification reports [8].

## 5. Conclusions

Considering the commercial implications, health risks associated with poisonous bamboo shoots, and nonavailability of optimized method for removal of TCC, we highlight the following for validating safe edible bamboo shoots prior to commercialization: (1) barcoding bamboo shoots using morphological descriptors and trnL-F intergenic spacer; (2) tagging the packed products with at least the genus name and beneficial nutritional components; (3) cogent tagging of genus* Dendrocalamus* with warning such as “bamboo shoots are injurious to health”; (4) refining the available edible bamboo shoots processing technique to minimize the residual quantity of TCC in the final products and normalizing the processing technique for stakeholders involved in the trade; (5) conserving germplasm, domesticating, and extensively farming *C. callosa* at high altitude of at least 1843 m from sea level to avoid extinction. 

## Supplementary Material

Table S1 shows qualitative and quantitative morphological descriptors. Table S2 shows list of primers use for performing random amplified polymorphic DNA analysis. Figure S1 shows RAPD gel profile using OPA-04 primer. Figure S2 shows sequence alignment entropy generated in BioEdit [19]. Table S3 shows principal component analysis for interrelatedness between nutritional attributes. Figure S3 shows comparative profile of point mutation at trnL-F intergenic spacer. Figure S4 shows test of homogeneity of substitution patterns between sequences.

## Figures and Tables

**Table 1:** Principal geographical coordinates, mean soil pH, and identified edible bamboo species.

GenBank accession/bamboo species	1	2	3	4	5	Voucher no.
KC013282/*Chimonobambusa callosa *	Leimaram	5.1 ± 0.3	24°44′	93°43′	1843 m (hill)	IBSD/WS/019
KC013285/*Bambusa cacharensis *	Arapti	5.8 ± 0.3	24°44′	93°51′	807 m (hill)	IBSD/WS/020
JX564900/*Bambusa manipureana *	Khong Khang	6.2 ± 0.1	24°21′	94°11′	240 m (valley)	IBSD/WS/008
JX564901/*Bambusa nutans *	Arapti	5.6 ± 0.4	24°44′	93°56′	226 m (valley)	IBSD/WS/023
JX507132/*Bambusa tulda *	Arapti	5.5 ± 0.1	24°44′	93°56′	226 m (valley)	IBSD/WS/022
JX507131/*Bambusa oliveriana *	Arapti	5.5 ± 0.3	24°44′	93°56′	728 m (hill)	IBSD/WS/010
JX564902/*Dendrocalamus giganteus *	Arapti	5.4 ± 0.2	24°44′	93°56′	803 m (hill)	IBSD/WS/001
JX564903/*Dendrocalamus hamiltonii *	Kwatha	6.2 ± 0.4	24°19′	94°16′	358 m (valley)	IBSD/WS/004
JX564904/*Dendrocalamus hookeri *	Arapti	4.9 ± 0.8	24°44′	93°56′	770 m (hill)	IBSD/WS/005
JX564905/*Dendrocalamus manipureanus *	Arapti	5.0 ± 0.4	24°21′	93°57′	769 m (hill)	IBSD/WS/002
JX507133/*Melocanna baccifera *	Lokchao	6.5 ± 0.4	24°20′	94°14′	497 m (hill)	IBSD/WS/018
JX507134/*Schizostachyum dullooa *	Arapti	5.0 ± 0.4	24°42′	93°57′	728 m (hill)	IBSD/WS/003
JX564906/*Bambusa* sp.	Arapti	5.2 ± 0.3	24°44′	93°56′	770 m (hill)	IBSD/WS/024
JX564907/*Bambusa* sp.	Andro	6.6 ± 0.3	24°47′	93°03′	803 m (hill)	IBSD/WS/007
KC013288/*Bambusa tuldoides *	Andro	6.4 ± 0.5	24°47′	93°03′	777 m (hill)	IBSD/WS/006

^1^Collection site, ^2^mean pH of the soil at the collection site in the months of July-August of 2009 to 2011, ^3^latitude (N), ^4^longitude (E), and ^5^altitude in meters showing either valley or hill where samples were collected.

**Table 2:** Total cyanide content (TCC) expressed in part per million (ppm) for different portions of bamboo shoots and their net antioxidant activity expressed as inhibition concentration (IC_50_) of DPPH.

Samples	Total cyanide content (ppm)*			IC_50_ (mg L^−1^)
	Tip	Middle	Base	
KC013282/*Chimonobambusa callosa *	300^a^	210^a^	199^a^	0.09^ab^
KC013285/*Bambusa cacharensis *	1533^d^	1221^f^	735^efgh^	0.58^h^
JX564900/*Bambusa manipureana *	1007^b^	515^b^	761^fgh^	0.46^fg^
JX564901/*Bambusa nutans *	1001^b^	624^c^	267^ab^	1.83^i^
JX507132/*Bambusa tulda *	1579^d^	1406^hi^	779^gh^	0.30^ef^
JX507131/*Bambusa oliveriana *	1280^c^	1079^e^	543^c^	0.57^gh^
JX564902/*Dendrocalamus giganteus *	2604^g^	2243^k^	920^i^	0.60^h^
JX564903/*Dendrocalamus hamiltonii *	1897^e^	766^d^	654^cdef^	0.14^bc^
JX564904/*Dendrocalamus hookeri *	1595^d^	1322^fgh^	360^b^	0.45^fg^
JX564905/*Dendrocalamus manipureanus *	1838^e^	1270^fg^	600^cd^	0.32^de^
JX507133/*Melocanna baccifera *	1548^d^	484^b^	216^a^	0.41^ef^
JX507134/*Schizostachyum dullooa *	1521^d^	1279^fg^	727^efgh^	0.61^h^
JX564906/*Bambusa* sp.	1582^d^	1354^gh^	669^defg^	0.24^cd^
JX564907/*Bambusa* sp.	2063^f^	1557^j^	635^cde^	0.25^cd^
KC013288/*Bambusa tuldoides *	2528^g^	1511^ij^	825^hi^	0.45^fg^
LSD (*P* ≤ 0.05)	85.78	106.53	109.31	0.11

*ppm = mg HCN equivalents/kg bamboo shoots and each value is the mean of three replicates for 2009, 2010, and 2011. The same letter(s) associated with mean values within a column is (are) not significantly different at *P ≤* 0.05 based on LSD.

**Table 3:** The data represented are obtained from atomic absorption spectrometry analysis showing the different mineral concentrations in milligram per 100 g dry weight (d.w) of bamboo shoots.

Samples	N	P	Ce	K	Na	Mg	Ca	Fe	Cu	Zn
KC013282/*C. callosa *	1153^m^	154^g^	4070.75^ab^	3377^e^	39^bc^	110^f^	63.43^gh^	9.25^abcd^	6.12^g^	7.2^de^
KC013285/*B. cacharensis *	815^g^	87^ab^	3157.82^a^	2093^abc^	45^cde^	74^ab^	29.60^bc^	13.15^def^	3.58^de^	5.63^bcd^
JX564900/*B. manipureana *	957^k^	94^bc^	5936.51^abc^	2343^bcd^	53^e^	175^i^	32.4^bcd^	16.5^f^	4.94^f^	12.80^i^
JX564901/*B. nutans *	825^gh^	104^cd^	5372.79^abc^	2090^abc^	32^b^	175^i^	53.5^fg^	11.6^cde^	4.16^e^	6.50^cde^
JX507132/*B. tulda *	721^c^	116^de^	3701.59^ab^	1453^abc^	51^de^	92^d^	42^de^	25.8^g^	3.19^cd^	15.0^j^
JX507131/*B. oliveriana *	763^e^	85^ab^	9972.34^cd^	2293^abc^	43^cd^	123^g^	60.95^fg^	10.1^bcd^	3.58^de^	4.88^bc^
JX564902/*D. giganteus *	673^a^	70^a^	7048.98^abcd^	3093^de^	22^a^	69^a^	37.9^cd^	7.83^abc^	2.47^bc^	10.7^gh^
JX564903/*D. hamiltonii *	747^d^	118^de^	4305.22^ab^	1910^abc^	50^de^	121^g^	24.4^ab^	16.82^f^	3.0^cd^	7.87^ef^
JX564904/*D. hookeri *	692^b^	75^a^	5697.05^abc^	2400^cd^	45^cde^	84^cd^	73.3^hi^	6.08^a^	2.64^bc^	3.03^a^
JX564905/*D. manipureanus *	894^i^	83^ab^	6784.58^abc^	3533^e^	42^cd^	85^cd^	14.98^a^	6.68^ab^	2.55^bc^	4.80^b^
JX507133/*M. baccifera *	1102^l^	135^f^	10126.98^cd^	1757^abc^	39^bc^	149^h^	51.88^ef^	13.37^ef^	1.96^ab^	11.93^hi^
JX507134/*S. dullooa *	837^h^	121^ef^	7098.87^abcd^	1310^a^	42^cd^	180^i^	56.95^fg^	14.42^ef^	1.55^a^	9.18^fg^
JX564906/*Bambusa* sp.	785^f^	73^a^	8306.12^bcd^	1373^ab^	32^b^	92^d^	40.1^cd^	11.5^cde^	2.54^bc^	11.33^g^
JX564907/*Bambusa *sp.	940^j^	129^ef^	11044.9^d^	1523^abc^	53e	112^f^	50.8^ef^	13^def^	7.85^h^	11.03^j^
KC013288/*B. tuldoides *	900^i^	122^ef^	4988.66^ab^	2060^abc^	31^b^	101^e^	78.15^j^	11.2^cde^	2.7^c^	11.0^h^
LSD (*P* = 0.05)	13.32	14.92	2858.21	873.06	8.51	8.28	10.17	3.48	0.64	1.55

The mean values are for replicates obtained in 2009, 2010, and 2011. The same letter(s) associated with mean values within column is (are) not significantly different at *P* ≤ 0.05 using LSD.
