# Supplementary material for: Grappling the High Altitude for Safe Edible Bamboo Shoots with Rich Nutritional Attributes and Escaping Cyanogenic Toxicity
Source: Biomed Res Int. 2013 Nov 19;2013:289285. doi: 10.1155/2013/289285 (PMC3852316; doi:10.1155/2013/289285)
Supplement: Supplementary file 1 — Table S1 shows qualitative and quantitative morphological descriptors. Table S2 shows list of primers use for performing random amplified polymorphic DNA analysis. Figure S1 shows RAPD gel profile using OPA-04 primer. Figure S2 shows sequence alignment entropy generated in BioEdit [19]. Table S3 shows principal component analysis for interrelatedness between nutritional attributes. Figure S3 shows comparative profile of point mutation at trnL-F intergenic spacer. Figure S4 shows test of homogeneity of substitution patterns between sequences. [file 289285.f1.docx]

**Supplementary information**


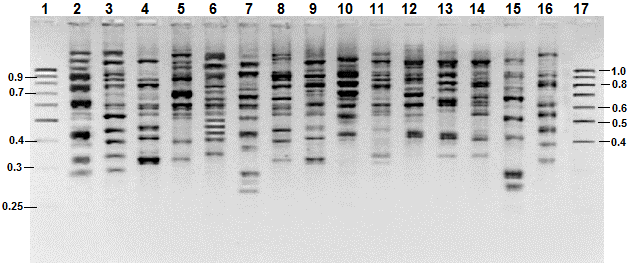


Figure S1: RAPD gel profile with OPA-04 primer showing the differences among the 15 edible bamboo species. Molecular ladder expressed in kilo base pair (lane-1 and lane-17), KC013282/*C. callosa* (lane-2)*,* KC013285/*B. cacharensis* (3), JX564900/*B. manipureana* (4), JX564901/*B. nutans* (5), JX507132/*B. tulda* (6), JX507131/*B. oliveriana* (7), JX564902/*D. giganteus* (8), JX564903/*D. hamiltonii* (9),JX564904/ *D. hookeri* (10), JX564905/ *D. manipureanus* (11), JX507133/ *M. baccifera* (12), JX507134/ *S. dullooa* (13), JX564906/ *Bambusa sp.* (14), JX564907/ *Bambusa sp*. (15) and KC013288/ *B. tuldoides* (16).


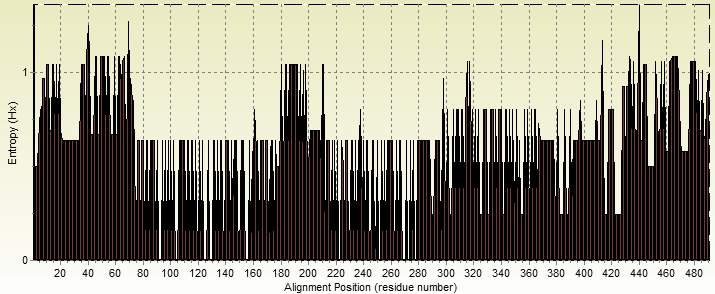


Figure S2: Sequence alignment entropy for 15 edible bamboo species generated in BioEdit [11], showing the overall differences among the sequences at the trnL‒F intergenic spacer.


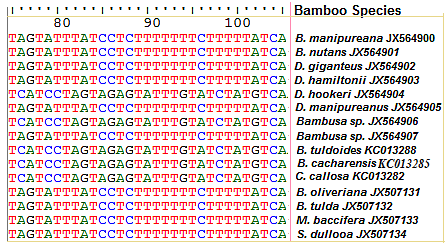


Figure S3: Comparative profile of point mutation at trnL‒F intergenic spacer of 15 edible bamboo species. Conserved nucleotide are highlighted in red while point mutated nucleotide are depicted in other colours. The analysis was performed in BioEdit (Windows TN version) [11].


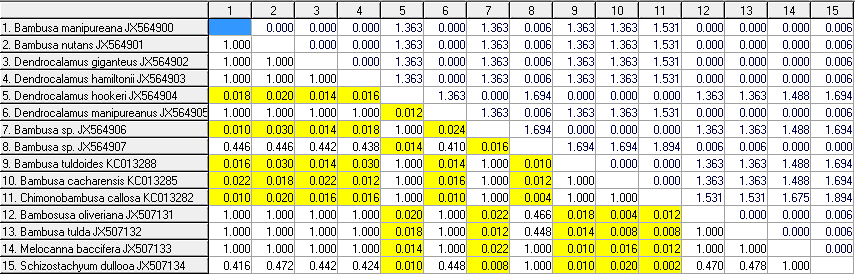


Figure S4: Test of the homogeneity of substitution patterns between sequences. The probability of rejecting the null hypothesis that sequences have evolved with the same pattern of substitution, as judged from the extent of differences in base composition biases between sequences (or disparity index test). A Monte Carlo Test (500 replicates) was used to estimate the *P*-values, which are shown below the diagonal. The *P*<0.05 are considered significant (marked with yellow highlights). The estimates of the disparity index per site are shown for each sequence pair above the diagonal. The analysis was performed in MEGA5 (Window TN version) [21].

| Table S1: Qualitative and quantitative morphological descriptors used for categorizing edible bamboo species are depicted | | | | | | | | | | | | | | | | | | | | | | | |
| --- | --- | --- | --- | --- | --- | --- | --- | --- | --- | --- | --- | --- | --- | --- | --- | --- | --- | --- | --- | --- | --- | --- | --- |
| Parameters | **Morphological descriptors** | | | | | | | | | | | | | | | | | | | | | | |
| **Quantitative descriptors** | Mean height | | Diameter | | | | | Thickness of wall | | | | | | Internode | | | Culm- sheath length | | | | | | Leaf breadth |
| **Qualitative descriptors**  **for culms** | ***Colour of culm*** | | | | | | | | | | | | | | | | | | | | | | |
|  | Grayish-green=0 | | Dull green=1 | | | | | Dark green=2 | | | | | | Glossy green=3 | | | | | Straw colour=4 | | | | |
|  | **Surface of the culm** | | | | | | | | | | | | | | | | | | | | | | |
|  | Pubescent=1 | | | | | | | Glabrous=2 | | | | | | | | | | | | White wax=3 | | | |
|  | ***Nodal ring*** | | | | | | | | | | | | | | | | | | | | | | |
|  | Absent =0 | Brown ring with conical spines =1 | | | | | | | White ring below the node=2 | | | | | | | Brown hairy ring=3 | | | | | | Marked with a thin ring=5 | |
|  | ***Nodal bud*** | | | | | | | | | | | | | | | | | | | | | | |
|  | Not prominent=0 | | | | | | | | Broad and ovate=1 | | | | | | | | | Oblique=3 | | | | | |
|  | ***Swollen node*** | | | | | | | | | | | | | | | | | | | | | | |
|  | Absent=0 | | | | | Present=1 | | | | | | | | | | | | | | | | | |
|  | ***Branching pattern*** | | | | | | | | | | | | | | | | | | | | | | |
|  | Starting from below node=0 | | | | | | Starting from above node=1 | | | | | | | | | | | | | | | | |
|  | ***Striations*** | | | | | | | | | | | | | | | | | | | | | | |
|  | Absent=0 | | | | | Present=1 | | | | | | | | | | | | | | | | | |
| **Qualitative descriptors**  **for culm sheath** | ***Hair colour*** | | | | | | | | | | | | | | | | | | | | | | |
|  | Brown=0 | | | | | Black=1 | | | | White=2 | | | | | | | | | | | | | |
|  | ***Hair distribution*** | | | | | | | | | | | | | | | | | | | | | | |
|  | Scanty=0 | | | | | Profuse=1 | | | | | | | | | | | | | | | | | |
|  | ***Imperfect blade*** | | | | | | | | | | | | | | | | | | | | | | |
|  | Erect=0 | | | | | Reflexed=1 | | | | | | | | | | | | | | | | | |
|  | ***Imperfect blade shape*** | | | | | | | | | | | | | | | | | | | | | | |
|  | Lanceolate=0 | | | Triangular=1 | | | | | | | | Subulate=2 | | | Ovate=3 | | | | | | Truncate=4 | | |
|  | ***Ligule margin*** | | | | | | | | | | | | | | | | | | | | | | |
|  | Fimbriate=0 | | | | Entire=1 | | | | | | | | Serrate=2 | | | | | | | | Dentate=3 | | |
|  | ***Auricle*** | | | | | | | | | | | | | | | | | | | | | | |
|  | Absent=0 | | | | Present=1 | | | | | | | | | | | | | | | | | | |
|  | ***Auricle continuous*** | | | | | | | | | | | | | | | | | | | | | | |
|  | Absent=0 | | | | Present=1 | | | | | | | | | | | | | | | | | | |
|  | ***Bristles*** | | | | | | | | | | | | | | | | | | | | | | |
|  | Absent=0 | | | | Present=1 | | | | | | | | | | | | | | | | | | |
|  | ***Auricle fringed*** | | | | | | | | | | | | | | | | | | | | | | |
|  | Absent=0 | | | | Present=1 | | | | | | | | | | | | | | | | | | |
|  | ***Sheath size*** | | | | | | | | | | | | | | | | | | | | | | |
|  | Small=0 | | | | Big=1 | | | | | | | | | | | | | | | | | | |
|  | ***Duration of culm sheath*** | | | | | | | | | | | | | | | | | | | | | | |
|  | Cauducous=0 | | | | Persistent=1 | | | | | | | | | | | | | | | | | | |
| **Qualitative descriptors**  **for leaves** | ***Leaf shape*** | | | | | | | | | | | | | | | | | | | | | | |
|  | Oblong-lanceolate=0 | | | | Linear-lanceolate=1 | | | | | | | | | | Broad-lanceolate=2 | | | | | | | | |
|  | ***Leaf blade margin*** | | | | | | | | | | | | | | | | | | | | | | |
|  | Absent=0 | | | | Scrabrous=1 | | | | | | Surface of adaxial side (glabrous)=0 | | | | | | | | | | Pubescent=1 | | |
|  | ***Surface of abaxial side*** | | | | | | | | | | | | | | | | | | | | | | |
|  | Glabrous=0 | | | | Pubescent=1 | | | | | | | | | | | | | | | | | | |
|  | ***Leaf sheath*** | | | | | | | | | | | | | | | | | | | | | | |
|  | Glabrous=0 | | | | Striate=1 | | | | | | Striate and callus present=2 | | | | | | | | | | Callus and keeled present=3 | | |
|  | ***Cilia on the margin*** | | | | | | | | | | | | | | | | | | | | | | |
|  | Absent=0 | | | | Present=1 | | | | | | | | | | | | | | | | | | |
|  | ***Auricle*** | | | | | | | | | | | | | | | | | | | | | | |
|  | Absent=0 | | | | Present=1 | | | | | | | | | | | | | | | | | | |
|  | ***Bristles*** | | | | | | | | | | | | | | | | | | | | | | |
|  | Absent=0 | | | | Present=1 | | | | | | | | | | | | | | | | | | |
|  | **Height of ligule** | | | | | | | | | | | | | | | | | | | | | | |
|  | Very short=0 | | | | Short=1 | | | | | | Long=2 | | | | Inconspicuous=3 | | | | | | Membranuous=4 | | |

Table S2: List of primers used for performing random amplified polymorphic DNA analysis

| **Name of primer** | **Sequence of the primer** | **Total number of amplified products** | **Number of polymorphic products** |
| --- | --- | --- | --- |
| OPA 04 | 5'-AATCGGGCTG-3' | 23 | 23 |
| OPA 11 | 5'-CAATCGCCGT-3' | 19 | 19 |
| OPA19 | 5'-TCTGTGCTGG-3' | 17 | 16 |
| OPA 17 | 5'-GACCGCTTGT-3' | 12 | 8 |
| OPA 20 | 5'-GTTGCGATCC-3' | 14 | 14 |
| OPN 11 | 5'-TCGCCGCAAA-3' | 18 | 18 |
| OPN 13 | 5'-AGCGTCACTC-3' | 20 | 20 |
| OPN 19 | 5'-GTCCGTACTG-3' | 8 | 8 |
| OPN 20 | 5'-GACCGACCCA-3' | 15 | 14 |
| **Total** |  | 146 | 140 |

Table S3: Principal component analysis parameters, interrelatedness between nutritional attributes for 12 edible bamboo species

| **PCA parameters** | | | | **Biochemical parameters** | | | | | | | | | | | |
| --- | --- | --- | --- | --- | --- | --- | --- | --- | --- | --- | --- | --- | --- | --- | --- |
|  | Eigen value | % of variance | % of Cumulation | **P** | **TCC** | **N** | **Mg** | **Na** | **K** | **Fe** | **Zn** | **AA** | **Ce** | **Ca** | **Cu** |
| PC1 | 3.86 | 32.19 | 32.19 | **0.785** | -0.54 | **0.54** | **0.59** | **0.70** | -0.64 | **0.73** | **0.50** | **0.36** | -0.01 | -0.09 | **0.68** |
| PC2 | 2.03 | 16.91 | 49.10 | -0.09 | 0.32 | **0.55** | **0.51** | 0.00 | **0.32** | -0.57 | **-**0.45 | -0.37 | **0.53** | 0.23 | **0.45** |
| PC3 | 1.74 | 14.50 | 63.59 | 0.22 | **0.67** | 0.01 | **-**0.26 | -0.27 | -0.37 | -0.06 | **0.41** | -0.13 | **0.55** | **0.57** | **0.34** |
| PC4 | 1.40 | 11.65 | 75.25 | -0.08 | 0.26 | 0.16 | -0.36 | 0.28 | 0.21 | -0.17 | -0.04 | **0.61** | **0.45** | -0.59 | 0.23 |

Values in bold indicate the most relevant characters (*r>0.30*) that contributed to most of the variation of the particular component at *P<0.05*.
